# The Hepatitis E Virus ORF3 Protein Regulates the Expression of Liver-Specific Genes by Modulating Localization of Hepatocyte Nuclear Factor 4

**DOI:** 10.1371/journal.pone.0022412

**Published:** 2011-07-20

**Authors:** Vivek Chandra, Prasida Holla, Dhrubaa Ghosh, Debarshi Chakrabarti, Muralidhara Padigaru, Shahid Jameel

**Affiliations:** 1 Virology Group, International Centre for Genetic Engineering and Biotechnology, Aruna Asaf Ali Marg, New Delhi, India; 2 Department of Biomarker Discovery, Piramal Life Sciences Limited, Mumbai, India; Saint Louis University, United States of America

## Abstract

The hepatitis E virus (HEV) is a small RNA virus and the cause of acute viral hepatitis E. The open reading frame 3 protein (pORF3) of HEV appears to be a pleiotropic regulatory protein that helps in the establishment, propagation and progression of viral infection. However, the global cellular effects of this protein remain to be explored. In the absence of traditional *in vitro* viral infection systems or efficient replicon systems, we made an adenovirus based ORF3 protein expression system to study its effects on host cell gene expression. We infected Huh7 hepatoma cells with recombinant adenoviruses expressing pORF3 and performed microarray-based gene expression analyses. Several genes down regulated in pORF3-expressing cells were found to be under regulation of the liver-enriched hepatocyte nuclear factor 4 (HNF4), which regulates hepatocyte-specific gene expression. While HNF4 localizes to the nucleus, its phosphorylation results in impaired nuclear localization of HNF4. Here we report that pORF3 increases HNF4 phosphorylation through the ERK and Akt kinases, which results in impaired nuclear translocation of HNF4 and subsequently the down modulation of HNF4-responsive genes in pORF3-expressing cells. We propose that modulation of several hepatocyte specific genes by pORF3 will create an environment favorable for viral replication and pathogenesis.

## Introduction

Hepatitis E virus (HEV) is the causative agent for hepatitis E, a waterborne disease that occurs sporadically and as focused outbreaks [1], [2]. It is endemic in large parts of Asia, Africa and Latin America from where large and small outbreaks and sporadic disease have also been reported [3]; about 2 billion people are estimated to live in areas where HEV infection is endemic [4]. While the infection is generally acute and self-limiting, up to 30% mortality has been reported following HEV infection during pregnancy [5]. Recently classified as a *Hepevirus* belonging to the family *Hepeviridae*, HEV is a non-enveloped virus with an ∼7.2 kb capped and polyadenylated positive sense RNA genome, which contains three open reading frames (ORFs). The *orf1* encodes the viral nonstructural polyprotein, *orf2* codes for the viral capsid protein, and *orf3* encodes a small protein that is proposed to optimize the host cell environment for viral replication.

The *orf*3 of HEV encodes a protein of 123 amino acids; a recent report proposed pORF3 to be translated from a bicistronic subgenomic RNA and to be 9 aa shorter [6]. Both the ORF3 proteins (123aa and 114aa) behave similarly in terms of their localization and functional effects in subgenomic expression systems [7]. The ORF3 protein is phosphorylated at a single serine residue by the cellular mitogen-activated protein kinase [8]. It contains two hydrophobic domains in its N-terminal half and two proline-rich regions in its C-terminal half, one of which contains the phosphorylated serine residue [8]. The other proline-rich region contains a PXXPXXP (where X is any other residue) motif that was shown to bind several proteins with src-homology 3 domains [9]. It was subsequently demonstrated that pORF3 activated the extracellularly regulated kinase, a member of the mitogen-activated protein kinase family of proteins, by binding and inhibiting its cognate phosphatase [10]. The ORF3 protein also attenuates the mitochondrial death pathway [11] and upregulates the expression of glycolytic pathway genes through the stabilization of hypoxia-inducible factor 1 [12]. We recently showed that pORF3 delays the movement of activated epidermal growth factor (EGF) receptors (EGFRs) and hepatocyte growth factor (HGF) receptors (c-Met) into the degradative compartment, which is likely to result in extended growth factor signaling from intracellular sites [7], [13]. This effect on EGFR movement was also found to retard the nuclear transport of phosphorylated signal transducer and activator of transcription 3 (pSTAT3), which attenuates the acute-phase response, a major inflammatory pathway in the liver [7].

All previous *in vitro* expression studies have shown pORF3 to regulate cellular processes and to have three broad roles. The first is the promotion of cell survival through activation of the ERK/MAPK signaling pathway, attenuation of the intrinsic death pathway, regulation of energy homeostasis, and increased growth factor signaling [7], [10], [11], [12]. Secondly, it attenuates host immune responses through reduced expression of acute-phase proteins and increased secretion of the immunosuppressive α1-microglobulin protein [7], [14]. Finally, recent reports have also suggested that pORF3 is required for virion morphogenesis and egress from infected cells [15]. Thus, pORF3 appears to be a regulatory protein required for the establishment, propagation, and progression of HEV infection. Indeed, it was found to be required for experimental HEV infection in nonhuman primates and pigs [16], [17]. For a global overview of its role in HEV biology, we performed transcript profiling of cultured Huh7 human hepatoma cells that were transduced with recombinant adenoviruses expressing the ORF3 protein. This analysis revealed a large-scale modulation of gene expression by the ORF3 protein. To understand the regulatory mechanisms we analyzed the list of modulated genes and found several down regulated genes to be known targets of hepatocyte nuclear factor 4 (HNF4), a liver-enriched transcription factor.

Hepatocyte nuclear factor 4, an orphan member of the nuclear receptor super family, regulates many genes that are preferentially expressed in the liver [18]. In addition to a relatively high level of expression in the liver, HNF4 mRNA and protein are also found in the kidney, intestine and colon and to a lesser extent in pancreas and stomach. The genes regulated by HNF4 play important roles in nutrient transport and metabolism, blood maintenance, immune function, liver differentiation and growth factors [18], [19], [20]. HNF4 binds DNA exclusively as a homodimer and is localized primarily in the nucleus. It contains a highly conserved Ser/Thr residue between the two zinc fingers that is adjacent to a nuclear localization signal (S78 in human HNF4). A recent report showed that phosphorylation of that site in HNF4 resulted in impaired nuclear localization and DNA binding, which would decrease the transcriptional effects of HNF4 [21]. HNF4 activity was also shown to be dependent upon ERK and Akt kinases [22], [23].

In this work we have carried out gene expression profiling of Huh7 liver cells expressing the HEV ORF3 protein and found several downregulated genes to be controlled by the liver-enriched transcription factor HNF4. We further showed that this was due to ERK- and Akt-mediated HNF4 phosphorylation, which reduced nuclear translocation of HNF4 and attenuated its transcriptional activity in ORF3-expressing cells. This study provides insights into the modulation of liver-specific gene expression during HEV infection.

## Materials and Methods

### Plasmids and antibodies

The expression vectors and monoclonal antibodies for pORF3 have been described earlier [7], [13]. The pShuttle-CMV-ORF3-EGFP and pShuttle-CMV-EGFP constructs were made by subcloning NheI-XbaI digested fragments from pORF3(114)-EGFP and pEGFP-N1 into XbaI digested pShuttle-CMV. The HEV replicon plasmid pSK-E2 was a kind gift from Suzanne Emerson (NIH, USA), the pShuttle-CMV and pAdEasy-1 plasmids, *E. coli* BJ5183 and *E. coli* DH10B were from S. Swaminathan (ICGEB, New Delhi, India) and the RFP-tagged Rab5 construct was provided by Satyajit Mayor (National Centre for Biological Sciences, India). The antibodies to phospho-ERK1, ERK1, EGFP, HIF-1α, tubulin and phospho-tyrosine were purchased from Santa Cruz Biotechnology (Santa Cruz, CA, USA). The antibodies to phospho-Akt, Akt, HNF4, PARP and STAT3 were purchased from Cell Signaling Technology (Beverly, MA, USA). Antibodies to phospho-serine were purchased from Sigma-Aldrich (St Louis, MO, USA). The HRP-conjugated secondary antibodies were from Calbiochem (San Diego, CA, USA) while the Alexa dye-conjugated secondary antibodies were from Molecular Probes (Eugene, OR, USA).

### Generation of recombinant adenoviral plasmids

The shuttle vector (1 µg) was linearized with *Pme*I and mixed with 0.1 µg of supercoiled pAdEasy-1 in a total volume of 6 µl. To this, 20 µl of electrocompetent *E.coli* BJ5183 cells were added, and electroporation was performed in 2 mm cuvettes at 2500 V, 200 Ώ and 25 mF in a Bio-Rad Gene Pulser electroporator. The cells were immediately placed in 1 ml of LB-media and grown at 37°C for 45 min. The cells were then pelleted down, resuspended in 100 µl of LB medium and plated on LB agar plates containing 50 µg/ml of kanamycin. After 16–20 hr of growth at 37°C, the smaller colonies (which usually represent the recombinants) were picked and grown in 2 ml of LB-media containing 50 µg/ml of kanamycin. Clones were first screened by analyzing their supercoiled sizes on agarose gels and compared to pAdEasy-1 controls. Those that appeared to contain inserts were then sequenced for final confirmation, and these were then electroporated into *E.coli* DH10B cells for large-scale amplification.

### Generation of recombinant adenoviruses

HEK-293 cells (ATCC) were plated in T25 flasks 24 hr prior to transfection. Recombinant adenoviral DNA was linearized with PacI and 4 µg was used together with 20 µl of Lipofectamine (Invitrogen, Carlsbad, CA, USA) in 500 µl of OptiMEM for transfection of each T25 flask. Transfected cells were monitored for GFP expression, and 7–10 days post-transfection cells were scrapped off and stored with culture media at −70°C. When required, cells with media were lysed using 4–6 cycles of freezing in liquid N2 and rapid thawing at 37°C. This lysate containing the virus was centrifuged at 12000xg for 10 min to remove cellular debris. The supernatant was stored at −70°C and used as the primary adenovirus stock. For amplification of recombinant adenoviruses, approximately 1.5×10^6^ HEK-293 cells were plated in a T25 flask 24 hr prior to infection. Cells were washed once with complete media and 1 ml of primary adenovirus stock was used to infect these cells. After 2 hr at 37°C, the primary adenovirus stock was removed, and 3 ml of growth medium was added. Three to four days later, viruses were harvested as described above. This process was repeated 4–6 times to get high titer virus.

### Reverse transcription polymerase chain reaction

Total RNA was isolated from cells using the Trizol reagent (Invitrogen, Carlsbad, CA, USA). Four µg RNA in a 25 µl reaction mixture was used for cDNA synthesis with oligo dT primers and Reverse Transcriptase (Promega, Madison, WI, USA) according to the supplier's protocol. Of this, 1 µl of the cDNA mixture was used as a template for PCR amplification of target genes. The PCR reactions were performed in a 50 µl volume containing 1x reaction buffer, 200 µM dNTPs, 10 pmol of each primer, and 1.25 units of Taq DNA polymerase (Real Biotech Corporation, Taipei, Taiwan), for 30–35 cycles of 94°C for 30 sec, 55°C for 30 sec, 72°C for 30 sec, and a final extension at 72°C for 5 min. Real-time PCR was performed using 1 µl of the cDNA mixture in a 20 µl volume containing 10 µl of 2X Power SYBR® Green PCR Master Mix (Applied Biosystems, CA, USA) and 5 pmol of each primer for 40 cycles of 95°C for 30 sec, 55°C for 30 sec, 72°C for 30 sec, and a final extension at 72°C for 5 min on a StepOnePlus™ Real-Time PCR System (Applied Biosystems, CA, USA). The threshold fluorescence level was automatically set by the software, and the threshold cycle (*CT*) was determined for each sample. Relative expression was calculated taking the control as 100%. Primer sequences were generated using the Primer Bank and were custom synthesized.

### Immunoprecipitation and western blotting

Cells were lysed with a buffer containing 20 mM Tris-HCl, pH 7.5, 150 mM NaCl, 1 mM EDTA, 1 mM EGTA, 1% Triton X-100 and a protease inhibitor cocktail (Roche, Mannheim, Germany). The clarified supernatant was quantified for protein concentration by the Bradford assay (Bio-Rad Laboratories, Hercules, USA). For immunoprecipitation, 1 mg of total proteins in 500 µl of lysis buffer were incubated with 20 µl of Protein A-agarose beads (GE Healthcare, Uppsala, Sweden) for 1 hr at 4°C. The pre-cleared lysate was then incubated with 2 µg of the antibody overnight at 4°C followed by 20 µl of Protein A-agarose beads for 2 hr at 4°C. After five washes in lysis buffer, the beads were boiled in Laemmli buffer, and the proteins separated by SDS-PAGE. For western blotting, proteins separated by SDS-PAGE were transferred to nitrocellulose membrane (Hybond ECL, GE Healthcare), the membrane blocked with Tris-buffered saline (TBS) containing 5% Blotto (Bio-Rad, Hercules, USA) for 1–2 hr at room temperature and washed with TBST (TBS containing 0.1% Tween-20). The membrane was then incubated overnight at 4°C with the primary antibody appropriately diluted in TBST-5% BSA, washed thrice for 10 min each with TBST and incubated with HRPO-linked secondary antibodies diluted in TBST-5% Blotto for 1 hr at RT. After washing as above, chemiluminescent detection of proteins was carried out using the Phototope Detection System (Cell Signaling Technology, Beverly, MA) according to the supplier's protocol.

### Replicon transfection and HEV infection

Plasmid pSK-E2 was linearized at a unique BglII site located immediately downstream of the HEV poly(A) tract, and capped transcripts were synthesized as previously described [24], [25]. Transcription mixtures were cooled on ice and then mixed with a liposome mixture consisting of 25 µl of DMRIE-C (1,2-dimyristyloxypropyl-3-dimethyl-hydroxy ethyl ammonium bromide and cholesterol; Invitrogen, Carlsbad, CA) in 1 ml of Opti-MEM. This was added to a T25 flask containing washed S10-3 cells at about 40% confluence. The S10-3 cells are a subclone of the Huh7 cell line and were a kind gift of Suzanne Emerson (NIH, USA). The flasks were incubated at 34.5°C for 6 hr, an additional 1 ml of Opti-MEM was added, and incubation was continued overnight. The cells were then trypsinized, split into two T25 flasks, and incubated in Dulbecco's modified Eagle's medium containing 10% FBS. On day 5 post-transfection, cells were again trypsinized and plated for immunostaining, which was performed on day 8 post-transfection. For virus infection, HEV was prepared as mentioned earlier [26]. For infection 0.2 ml of virion mix was added to the cells, and incubated at 37°C in a 5% CO_2_ atmosphere for 2 hr, after which the liquid was replaced with 1 ml of growth medium containing antibiotics. On day 3 post-infection, cells were again trypsinized and plated for immunostaining, which was performed on day 5 post-infection.

### Microscopy

To study the localization of pORF3, recombinant adenovirus infected Huh7 cells were transiently co-transfected with the appropriate expression vectors. After 48 hr the cells were fixed using 2% paraformaldehyde for 20 min and imaged using a confocal microscope (Nikon A-1R). For the staining of endogenous HNF4 and HEV replicon expressed pORF3, cells were fixed with 2% paraformaldehyde for 20 min, permeablized at room temperature with 0.4% Triton X-100 for 15 min and stained using the appropriate primary and secondary antibodies.

### Microarray

Huh7 cell were infected with ORF3-EGFP and control EGFP expressing recombinant adenoviruses. After 48 hr of infection, cells were harvested and RNA was isolated from both the test and control samples using Trizol reagent (Invitrogen, USA). The extracted RNAs were further purified using the RNeasy mini kit (Qiagen,USA) according to the manufacturer's protocol. Of these, 10–12 µg purified control and test RNAs were used as templates for cDNA synthesis, using amino-allyl dNTPs, with the ChipShot Indirect Labeling System (Promega, USA) according to the supplier's protocol. Purification of amino-allyl cDNA and post labeling with Cyanine 3 or 5 (Amersham, Little Chalfont, UK) was carried out with the same system. RNA extracted from control cells was labeled with Cyanine 3 (Cy3) and that from test cells was labeled with Cyanine 5 (Cy5). The samples were then lyophilized, resuspended in hybridization buffer (Pronto™ Universal Hybridization kit, Corning) and incubated at 95°C for 5 min. The labeled cDNAs from control and test samples were then mixed, centrifuged at high speed for 5 min and hybridized on 35K_operon oligo_ver4.0 (Nicholas Piramal, Mumbai, India). The slides were given pre- and post- hybridization treatment using the Pronto Universal Hybridization kit (Corning) according to the supplier's instructions. Hybridization was carried out in a Hybstation (Genomic Solutions) hybridization chamber according to manufacturer's instruction. The hybridization profile used was 42°C for 6 hr, 35°C for 6 hr and 30°C for 6 hr. Scanning and data analysis were performed as described elsewhere [27]. Gene Ontology and transcription factor analysis were done with FatiGO, a functional enrichment software. The raw microarray data has been deposited in a MIAME compliant database, as detailed on the MGED Society website http://www.mged.org/Workgroups/MIAME/miame.html. The GEO accession number for this data is GSE23412.

## Results

### Generation and characterization of ORF3 recombinant adenovirus

Recombinant adenoviruses expressing the *orf3-egfp* or *egfp* were constructed as described in Materials and Methods. Viral stock normalization was done by RT-PCR for the adenovirus hexon gene. The infectious titer of the adenovirus stock is usually determined by plaque assay. But, that has several limitations including the length of time required to perform the assay. The assay based on RT-PCR of adenovirus mRNA is rapid, sensitive, and reproducible. Importantly, there is a linear correlation between the titer determined by real-time PCR and the infectious titer determined by a plaque assay [28]. Further, previous work from our lab suggested a cell survival role for the HEV ORF3 protein [11]. A plaque assay based on cell death may not give an accurate measurement of the infectious virus present. We therefore used the RT-PCR based assay to normalize the recombinant adenovirus titers.

**Figure 1:**
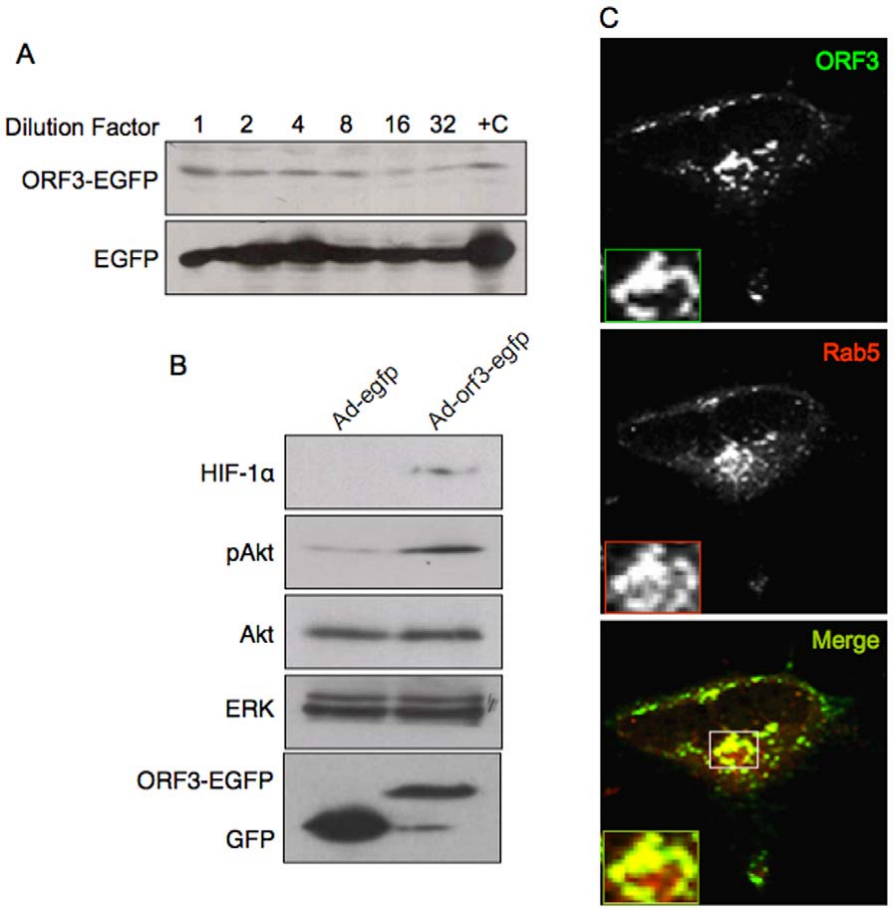
Functional characterization of the ORF3 protein expressed from adenoviral recombinants. (A) Huh7 cells were infected with different dilutions of the Ad-orf3-egfp and Ad-egfp recombinant adenoviruses. After 36 hr, the cells were lysed and the lysates western blotted with anti-GFP antibodies. Lane +C contains positive control lysate of Huh7 cells transfected to express ORF3-EGFP or EGFP. No signal was observed in uninfected cells (not shown). (B) Huh7 cells were infected with the Ad-orf3-egfp or Ad-egfp recombinant adenoviruses. After 48 hr, the cells were harvested and cell lysates were western blotted with anti-HIF1α, anti-pAkt or anti-Akt antibodies. Western blotting with anti-ERK and anti-EGFP served as loading and expression controls, respectively. (C) Huh-7 cells were infected with ORF3-EGFP expressing recombinant adenovirus. After 24 hr, the cells were transfected with a Rab5-RFP expression construct, and 36 hr later, the cells were imaged by confocal microscopy. Monochrome and pseudocolored details of the boxed regions are shown and coded according to the color assigned for each entity.

Human hepatoma Huh7 cells were infected with different dilutions of crude stocks of Ad-orf3-egfp and Ad-egfp viruses and western blotting was used to test expression of the relevant proteins (Fig. 1A). To ensure that pORF3 expressed from infection with recombinant adenoviruses was functionally similar to that expressed following plasmid transfection, we checked its effects on HIF-1α and Akt. Analogous to earlier studies [12], higher levels of HIF-1α and phospho-Akt (but not total Akt) were observed in Huh7 cells transduced with Ad-orf3-egfp compared to control Ad-egfp (Fig. 1B). Our earlier studies using plasmid or replicon based pORF3 expression showed this protein to localize to early and recycling endosomes [7]. Huh7 cells were infected with the Ad-orf3-egfp virus and 24 hr post-infection the cells were transfected with a Rab5-RFP expression construct. As earlier, we found that pORF3 expressed by the adenoviral recombinant also localized to Rab5 positive compartments (Fig. 1C). These results support the use of recombinant adenoviruses for subsequent studies.

### Global modulation of gene expression by pORF3 in hepatoma cells

**Figure 2:**
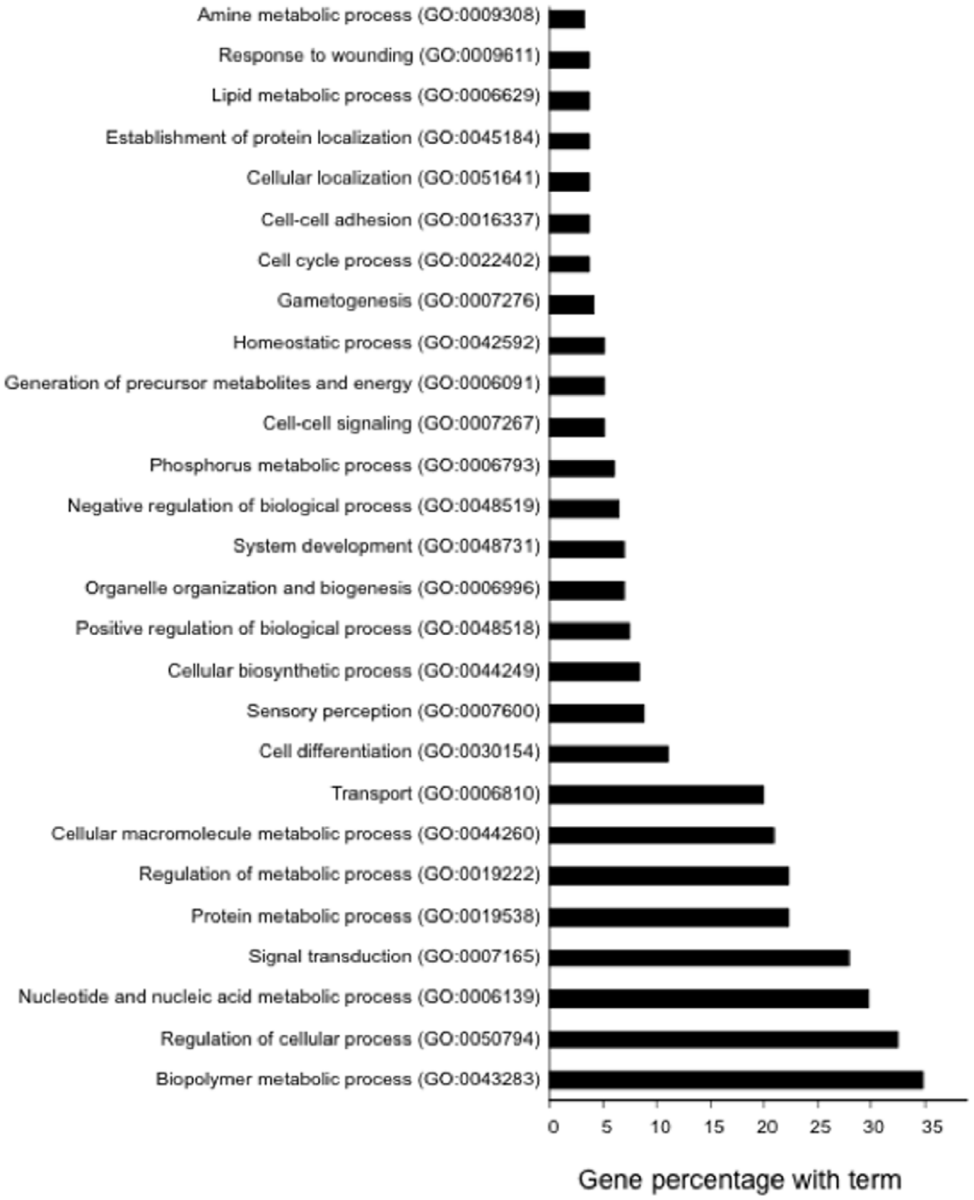
The ORF3 protein modulates several host processes. The modulated genes were analyzed with the FatiGO software and were divided into several clusters on the basis of GO terms. Only those GO classes with ≥3% genes are shown.

The human genome contains ∼25,000 predicted protein-coding genes [29]. Viruses can cause large-scale modulation of host genes to optimize their own replication and pathogenesis; such changes in host gene expression are analyzed using high throughput platforms such as microarrays [30]. We infected Huh7 cells with the Ad-orf3-egfp and Ad-egfp recombinant adenoviruses and purified the cellular RNA. The labeled cDNA synthesis, hybridization to a 35K_operon oligo_ver4.0 Human Array (Nicholas Piramal, India), scanning and data analysis were performed as described in Materials and Methods. Fold-change analysis showed a considerable difference in the gene expression profile of the ORF3-expressing cells compared to control cells. To make the data more robust we repeated this experiment three times and considered only those genes that showed similar profiles in all three experiments (Fig. S1). We found 221 genes to be down regulated and 115 genes to be upregulated in ORF3 expressing cells (Tables S1 and S2). One of the goals of microarray data analysis is to cluster genes with similar functional profiles to make meaningful biological inferences. FatiGO is a web tool that carries out data mining using Gene Ontology (GO) for DNA microarray data. The modulated genes were analyzed with this software to understand the biological pathways modulated by pORF3; these were divided into several clusters on the basis of GO terms (Fig. 2). This clustering indicated that pORF3 modulated several biological processes, especially metabolic pathways in the host cell.

### The ORF3 protein down regulates hepatocyte nuclear factor 4 dependent genes by interfering with its nuclear translocation

**Figure 3:**
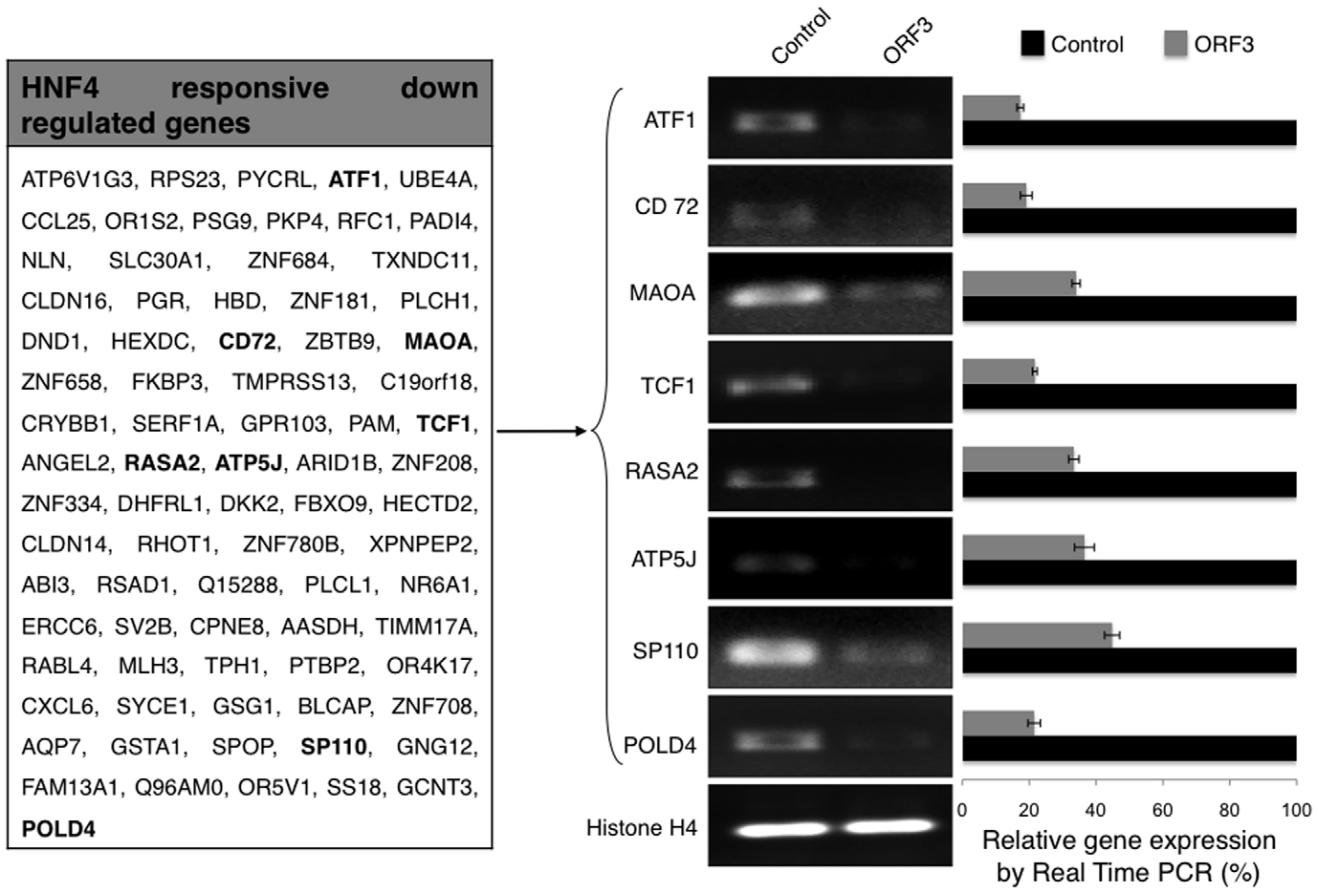
The ORF3 protein modulates HNF4 responsive genes. The list shows all HNF4 responsive genes that were found to be down regulated in Ad-orf3-egfp infected Huh7 cells compared to Ad-egfp infected cells. The genes shown in bold were validated by RT-PCR and real-time RT-PCR. For that, Huh7 cells were infected with the recombinant adenoviruses, and 48 hr post-infection, RNA was prepared and RT-PCR and real-time RT-PCR were carried out using primers shown in Table S3. Histone H4 served as a loading control for RNA. For real time RT-PCR relative expression was calculated taking the control as 100%.

Gene expression is modulated by transcription factors that occupy specific binding sites within the promoter/enhancer elements of the gene. To check if any major transcription factor was responsible for pORF3-mediated modulation of host genes, we analyzed the modulated gene lists using FatiGO [31]. Our analysis found 79 (35.7%) of the 221 down regulated genes to be known targets of hepatocyte nuclear factor 4 (HNF4), a liver-enriched transcription factor. To validate the effects of pORF3 on HNF4-modulated genes, we carried out RT-PCR and real-time RT-PCR analysis for eight such randomly chosen genes using gene-specific primers (Table S3). As expected, both analyses showed decreased expression of ATF1, CD72, MAOA, TCF1, RASA2, ATP5J, SP110 and POLD4 (Fig. 3).

**Figure 4:**
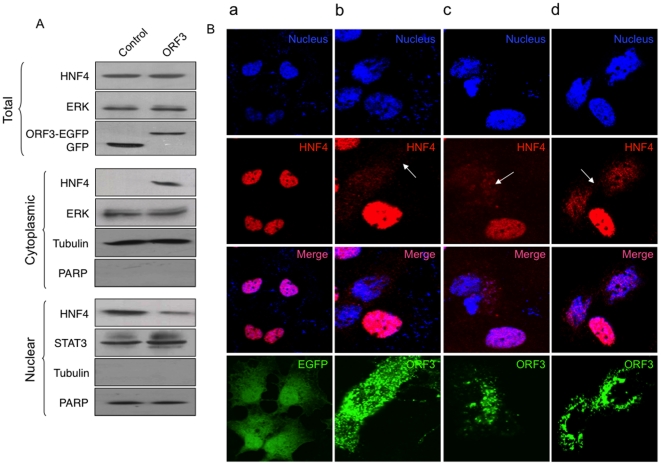
The ORF3 protein interferes with the nuclear localization of HNF4. (A) Huh7 cell were infected with the control and ORF3-expressing recombinant adenoviruses. After 48 hr, total, cytoplasmic and nuclear lysates were prepared and western blotted with anti-HNF4. ERK and STAT3 served as loading controls for cytoplasmic and nuclear fractions, respectively. The cytoplasmic and nuclear lysates were also checked for tubulin and PARP to confirm their purity. (B) Huh7 cells were transfected with (a) pEGFP-N1 expression vector, (b) the pORF3-EGFP expression vector or (c) *in vitro* synthesized capped HEV replicon RNA. In panel (d) cells were infected with HEV prepared by replicon transfection as described. The cells were fixed and stained with anti-HNF4 and Alexa568-conjugated secondary antibodies, and DAPI (for nuclei). In (c) and (d) the cells were also stained with anti-ORF3 and Alexa488-conjugated secondary antibodies. The fixed cells were then imaged on a Nikon AR-1 confocal microscope. The EGFP or ORF3 protein, DAPI and HNF4 signals were pseudocolored green, blue and red, respectively. The merged pictures of single confocal planes are shown.

The HNF4 transcription factor regulates many liver-specific genes [18]. To check the cellular levels of HNF4, we infected Huh7 cells with the recombinant adenoviruses, prepared total, cytoplasmic and nuclear lysates, and carried out Western blotting with anti-HNF4 antibodies. While no difference was observed in the total HNF4 protein levels, higher cytoplasmic and lower nuclear levels of HNF4 were found in cells expressing pORF3 (Fig. 4A), suggesting that it might interfere with the nuclear localization of HNF4. The subcellular localization of HNF4 was also checked by confocal microscopy. While in control cells, HNF4 was quantitatively localized to the nucleus (Fig. 4Ba), in ORF3-expressing cells it was predominantly found in the cytoplasm (Fig. 4Bb,c,d). This subcellular localization of HNF4 was identical in cells expressing either the ORF3-EGFP fusion protein from a plasmid vector (Fig. 4Bb) or the native ORF3 protein from a transfected HEV replicon (Fig. 4Bc). Similar HNF4 and pORF3 localization was also observed in cells infected with HEV (Fig. 4Bd). These results suggest that pORF3 interferes with the nuclear localization of HNF4, which in turn is responsible for the reduced expression of HNF4 responsive genes.

### The pORF3-mediated phosphorylation of HNF4 is responsible for its reduced nuclear translocation and transcriptional activity

**Figure 5:**
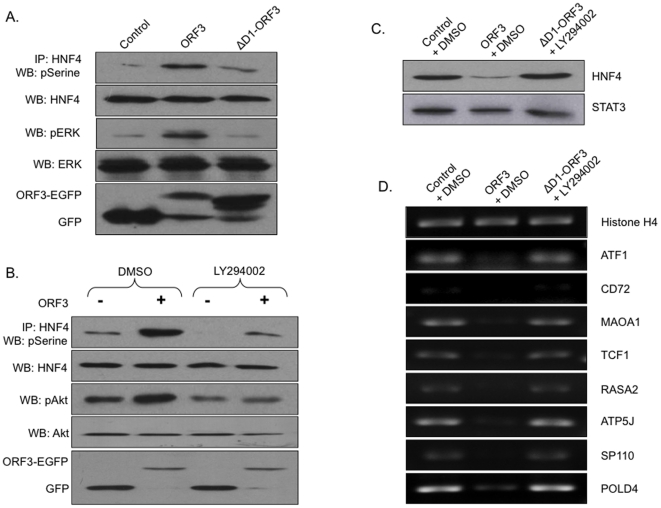
The ORF3 protein modulates HNF4 phosphorylation. (A) Huh7 cells were transfected to express EGFP (control) or the ORF3-EGFP or ΔD1-ORF3-EGFP fusion protein, and cells were harvested 48 hr post-infection. Cell lysates containing equal amounts of total protein were immunoprecipitated with anti-HNF4 and then western blotted with anti-pSerine antibody. Normalized lysates were western blotted with anti-HNF4 and anti-GFP antibodies for loading and expression controls, respectively. Normalized lysates were also western blotted with anti-pERK and anti-ERK to show the effect of pORF3 on ERK activation. (B) Huh7 cells were infected with ORF3 expressing (+) and control (−) recombinant adenoviruses. At 36 hr post infection, the cells were treated with either 50 µM LY294002 or an equal volume of DMSO for 12 hr. Cells were harvested 48 hr post-infection and lysates containing equal amounts of total protein were immunoprecipitated with anti-HNF4 and followed by western blotting with anti-pSerine antibody. Normalized lysates were western blotted with anti-HNF4 and anti-GFP antibodies for loading and expression controls, respectively. Normalized lysates were also western blotted with anti-pAkt and anti-Akt to show the effect of pORF3 on Akt activation. (C) Huh7 cells were transfected to express EGFP (control) or the ORF3-EGFP or ΔD1-ORF3-EGFP fusion protein. At 36 hr post-transfection, the cells transfected with ΔD1-ORF3-EGFP expressing plasmid were treated with 50 µM LY294002 and others were treated with an equal volume of DMSO for 12 hr. Nuclear lysates were prepared and western blotted with anti-HNF4. STAT3 served as a loading control. (D) Huh7 cells were transfected and treated as described in (C). RT-PCR analysis was performed as mentioned earlier. Histone H4 served as a loading control for RNA.

The ORF3 protein is likely to modulate the subcellular localization of HNF4 in two possible ways. It could interact with HNF4 and trap it in the cytoplasm or endosomal compartments where pORF3 is mainly present. Alternatively, pORF3 could interfere with the regulatory mechanism responsible for the nuclear localization of HNF4. A recent report suggested that phosphorylation of a highly conserved serine (Ser78) in HNF4 resulted in its impaired nuclear localization [21]. Further, HNF4 activity was shown to depend upon ERK and Akt kinases [22], [23]. Our previous studies have shown that ERK and Akt are activated in ORF3-expressing cells as well [10], [12]. To test for these alternative pathways, Huh7 cells were transfected with plasmids expressing ORF3-EGFP or EGFP (control). The cell lysates were immunoprecipitated with anti-HNF4 followed by western blotting with either anti-phosphoSerine (for phospho-HNF4) or anti-EGFP (for pORF3) antibodies. We did not find co-immunoprecipitation of pORF3 and HNF4 (data not shown) but found significant increase in phosphorylated HNF4 levels in ORF3-expressing cells (Fig. 5A). It was not possible to demonstrate these effects of the ORF3 protein on HNF4 phosphorylation with the full-length replicon due to its poor replication efficiency and a delay of 3-4 weeks in producing measurable virions in culture. We have earlier shown the hydrophobic domain 1 of pORF3 to be responsible for ERK activation. Here we found reduced HNF4 phosphorylation in cells expressing the domain 1-deleted (ΔD1) ORF3 protein compared to the wild type protein (Fig. 5A). Blocking of Akt activation with its pharmacological inhibitor LY294002 also resulted in attenuation of pORF3-mediated HNF4 phosphorylation (Fig. 5B). The use of a dominant negative mutant of the catalytic subunit of phosphoinositide 3-kinase (p110DN) did not show any measurable effects on pORF3-mediated HNF4 phosphorylation (not shown). To confirm that the pORF3-mediated effect on HNF4 nuclear localization was due to its phosphorylation, we blocked both the kinases by treating ΔD1-ORF3 expressing cells with LY294002, and checked the nuclear levels and transcription factor activity of HNF4. In these cells, the nuclear levels of HNF4 were comparable to control cells (Fig. 5C), and the transcriptional repression of HNF4-responsive genes was also relieved (Fig. 5D). Together these results confirm that HEV pORF3 increases HNF4 phosphorylation through the activation of ERK and Akt pathways. This is responsible for reduced nuclear localization and transcription factor activity of HNF4.

## Discussion

The host-pathogen interface represents the ultimate battleground for survival. Considerable progress has been made in understanding the roles that viral and cellular proteins play in determining the host susceptibility to infection and disease. Like most viruses, HEV also modulates the host cell machinery for its replication and pathogenesis. The HEV genome encodes the ORF1, ORF2 and ORF3 proteins, of which the ORF3 protein appears to possess host cell regulatory properties [32], [33]. Based on previous studies three broad roles are predicted for the ORF3 protein in HEV pathogenesis. The first is promotion of cell survival through ERK activation [10], attenuation of the intrinsic death pathway [11] and regulation of energy homeostasis [12]. The second is to down regulate host innate immune responses through reduced expression of acute phase proteins [7] and increased secretion of α1-microglobulin, an immunosuppressive protein [14]. Finally, it is shown to have a role in virion morphogenesis and viral release from infected cells [15]. Thus, pORF3 appears to be a pleiotropic regulatory protein that helps in the establishment and propagation of HEV infection by modulating multiple pathways. We therefore planned to get a global overview of the effects of ORF3 expression on liver cells. Due to the lack of traditional *in vitro* viral infection systems or efficient replicon systems, which can be used to study the effects of HEV proteins on cellular processes, our previous strategy was mainly based on subgenomic expression of pORF3.

In the last decade, recombinant adenovirus vectors have been used for a variety of purposes, including gene transfer *in vitro* and *in vivo*. The ability of recombinant adenoviruses to transduce a broad variety of cell types with high efficiency, regardless of the proliferative state of the target cells, as well as their relative ease of manipulation and preparation, makes them very powerful tools in mammalian gene expression studies. We therefore constructed recombinant adenoviruses expressing the ORF3 protein to check its importance for HEV infection, genome replication and pathogenesis with a reasonable virus based delivery system. Our earlier studies showed that pORF3 localizes to early and recycling endosomal compartments [7]. The adenovirus-expressed pORF3 faithfully localized to Rab5 positive compartments, as is the case with pORF3 expressed from HEV replicons or subgenomic expression vectors. We also checked its functional effect on some of the host proteins and found similar effects following expression using the adenovirus background. These finding support the use of recombinant adenovirus for gene expression analysis. To enhance our understanding of the role of pORF3 in HEV biology, hepatoma cells were infected with ORF3 or control recombinant adenoviruses and microarray-based gene expression analysis was carried out. Of the 25,500 genes that were analyzed, 115 genes were upregulated and 221 genes down regulated consistently in ORF3-expressing cells. All these genes were functionally grouped using the FatiGO software and were found to be grouped in different gene ontology (GO) functional groups [31]. The ORF3 protein was found to affect several pathways, especially the metabolic and signaling pathways. Transcription factor analyses showed a large number of genes driven by HNF4 to be down regulated in ORF3-expressing cells.

The HNF4 transcription factor is expressed mainly in the liver and plays important roles in nutrient transport and metabolism, blood maintenance, immune function, liver differentiation and expression of growth factors [18], [19], [20]. The HNF4 protein is expressed early in embryonic life, long before liver development and mice deleted for this gene die *in utero* between days 9.5-10.5, and exhibit impaired gastrulation [20]. In the adult liver, HNF4 is required for its proper function. Other hepatotropic viruses like HBV and HCV also modulate HNF4 for their efficient replication and pathogenesis [34], [35]. An earlier study from our group also found lower plasma levels of albumin and transthyretin in HEV infected patients [36]; the genes for these are major targets of HNF4. On transcriptional analysis, several of the down regulated genes were found to be regulated by HNF4, and this was also observed in nuclear levels of the HNF4 protein. Our microscopy data clearly proved that in a replicon expression model as well as in a virus infection system, pORF3 modulated the nuclear localization of HNF4. A recent report suggested that phosphorylation of a highly conserved serine (Ser78) in HNF4 resulted in its impaired nuclear localization [21], and HNF4 activity was dependent upon ERK and Akt kinases [22], [23]. Our previous studies have shown that ERK and Akt are also activated in ORF3-expressing cells [10], [12]. To test for these alternative pathways, we used the hydrophobic domain 1 deleted pORF3 which is unable to activate ERK and the pharmacological inhibitor LY294002 that blocks Akt activation, and found that this inhibition significantly reduces pORF3-mediated phosphorylation of HNF4. However, blocking of either pathway alone was not sufficient to completely revert this effect. Our results indicate that pORF3 regulates several hepatotropic proteins by inducing the phosphorylation of HNF4, which would in turn reduce its translocation to the nucleus and attenuate its transcription factor activity.

We have demonstrated large-scale modulation of HNF4-mediated gene expression as a consequence of ORF3 expression in Huh7 cells. One shortcoming of our work is its inability to directly demonstrate whether any of these changes in Huh7 cells actually affected HEV replication. An *orf3*-deleted HEV replicon replicates just as well as the wild type replicon when transfected into Huh7 cells [25]. However, the ORF3 protein is required for successful infection, virus replication and liver injury in macaques [16]. Thus, we are not likely to see the effects of the ORF3 protein on HEV replication in this Huh7 culture model. The macaque model appears to be the only one in which this could be done, and that is beyond the scope of this study.

Collectively our data provide a novel example of a viral protein that blocks the expression of several liver-specific genes by modulating the nuclear localization of HNF4. By regulating HNF4 localization and thereby its transcription factor activity, HEV is likely to modulate the expression of several hepatocyte genes. This could contribute positively to viral replication and pathogenesis.

## Supporting Information

Figure S1 **Heat map of cellular genes modulated in ORF3 expressing cells.** A heat map of genes modulated in the ORF3 expressing cells is shown for all three technical replicates, which was generated using software TM4 MultiExperiment Viewer. Gene list is in the same order as given in Table S1 (for down regulated genes) and Table S2 (for upregulated genes). The log2 transformed fold-change values were loaded and genes were sorted in load order to prepare the heat map. Color bar represents log2 transformed fold change values from −3.15 to + 3.15.
(TIF)

Table S1 **Cellular genes down regulated in ORF3-expressing cells.**
(DOC)

Table S2 **Cellular genes upregulated in ORF3-expressing cells.**
(DOC)

Table S3 **RT-PCR primers.**
(DOC)
