# Supplementary figures and images for: The Hepatitis E Virus ORF3 Protein Regulates the Expression of Liver-Specific Genes by Modulating Localization of Hepatocyte Nuclear Factor 4
Source: PLoS One. 2011 Jul 20;6(7):e22412. doi: 10.1371/journal.pone.0022412 (PMC3140526; doi:10.1371/journal.pone.0022412)

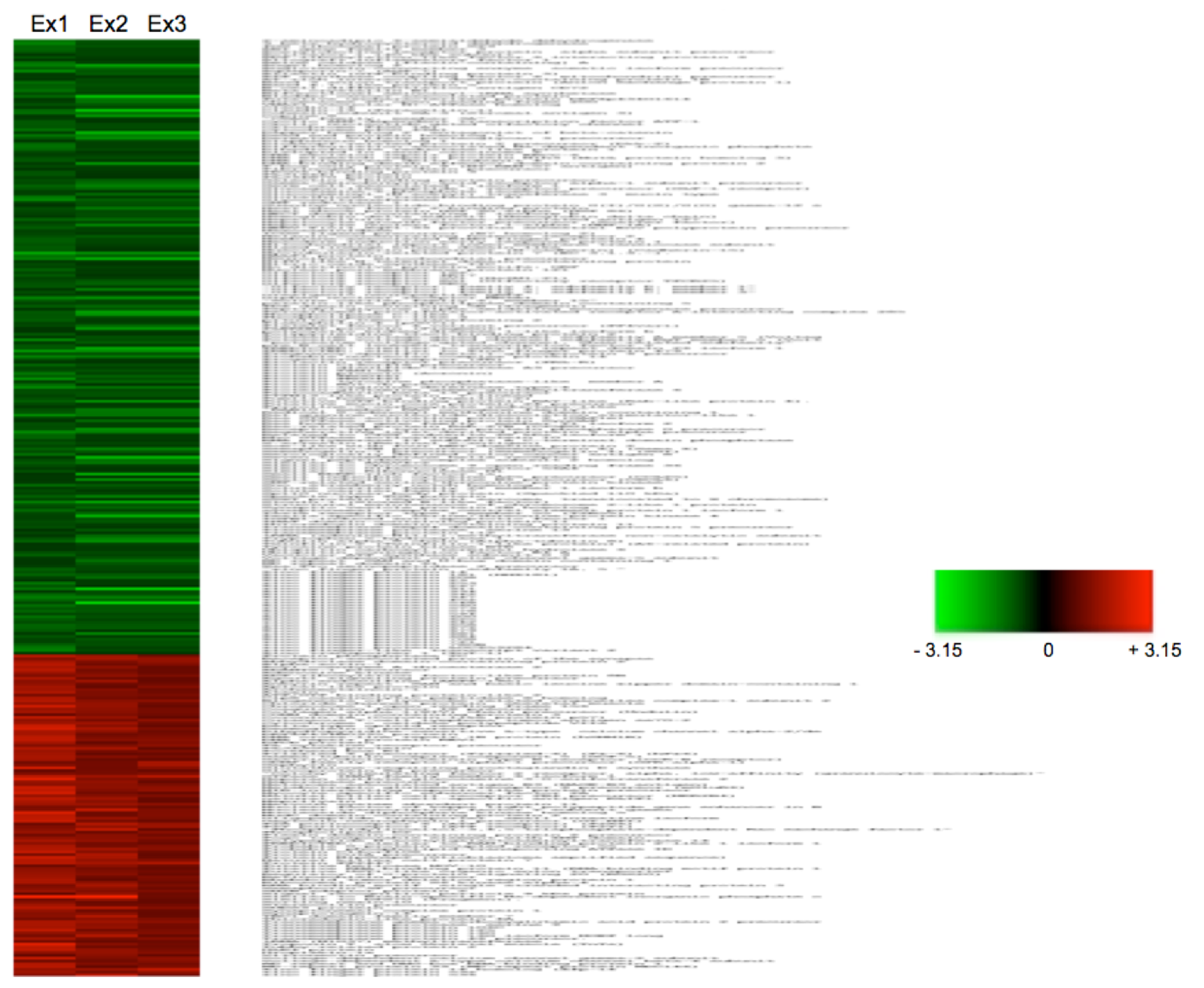

Supplement: Figure S1 — Heat map of cellular genes modulated in ORF3 expressing cells. A heat map of genes modulated in the ORF3 expressing cells is shown for all three technical replicates, which was generated using software TM4 MultiExperiment Viewer. Gene list is in the same order as given in Table S1 (for down regulated genes) and Table S2 (for upregulated genes). The log2 transformed fold-change values were loaded and genes were sorted in load order to prepare the heat map. Color bar represents log2 transformed fold change values from −3.15 to + 3.15. (TIF) [file pone.0022412.s001.tif]
