# Supplementary material for: The Hepatitis E Virus ORF3 Protein Regulates the Expression of Liver-Specific Genes by Modulating Localization of Hepatocyte Nuclear Factor 4
Source: PLoS One. 2011 Jul 20;6(7):e22412. doi: 10.1371/journal.pone.0022412 (PMC3140526; doi:10.1371/journal.pone.0022412)
Supplement: Table S1 — Cellular genes down regulated in ORF3-expressing cells. (DOC) [file pone.0022412.s002.doc]

**Table S1. Cellular genes down regulated in ORF3-expressing cells**

| Gene Name | Gene Symbol |
| --- | --- |
| Breakpoint cluster region protein | NP_997357.1|BCR |
| 2-aminoadipic 6-semialdehyde dehydrogenase | AASDH |
| 3 beta-hydroxysteroid dehydrogenase | Q9UD07 |
| 40S ribosomal protein S23 | RPS23 |
| ABI gene family member 3 | ABI3 |
| Acetylcholine receptor protein alpha subunit precursor | CHRNA1 |
| Activator 1 140 kDa subunit | RFC1 |
| ADP-ribosylation-like factor 6 interacting protein 6 | ARL6IP6 |
| Allograft inflammatory factor 1 |  |
| Amine oxidase [flavin-containing] A | MAOA |
| Angel homolog 2 | ANGEL2 |
| Angiotensin-converting enzyme somatic isoform precursor | ACE |
| Aquaporin-7 (AQP-7) | AQP7 |
| Atlastin (GTP-binding protein 3) | SPG3A |
| ATP synthase coupling factor 6 mitochondrial precursor | ATP5J |
| AT-rich interactive domain-containing protein 1B | ARID1B |
| Band 3 anion transport protein (Anion exchange protein 1) | SLC4A1 |
| Band 4.1-like protein 5 | EPB41L5 |
| B-cell differentiation antigen CD72 | CD72 |
| Beta crystallin B1. | CRYBB1 |
| Bifunctional aminoacyl-tRNA synthetase | EPRS |
| Bladder cancer-associated protein | BLCAP |
| Putative uncharacterized protein DKFZp434O1614 | Q8NDA5 |
| Chromosome 20 open reading frame 200 | C20orf200 |
| VMA21 vacuolar H+-ATPase homolog | VMA21 |
| Claudin-14 | CLDN14 |
| Claudin-16 (Paracellin-1) | CLDN16 |
| Cohesin subunit SA-3 (Stromal antigen 3) | STAG3 |
| Copine-8 | CPNE8 |
| CSAG family, member 3A |  |
| Cyclic AMP-dependent transcription factor ATF-1 | ATF1 |
| Cyclic nucleotide-gated olfactory channel | CNGA2 |
| Cytochrome P450 1A1 | CP1A1 |
| Cytochrome p450 46A1 | CYP46A1 |
| Dapper homolog 2 antagonist of beta-catenin | DACT2 |
| Dead end protein homolog 1 | DND1 |
| Dermatan sulfate proteoglycan 3 precursor | DSPG3 |
| Diaphanous homolog 1 |  |
| Dickkopf-related protein 2 precursor (Dkk-2) | DKK2 |
| Differentiation-associated Na-dependent inorganic phosphate | SLC17A6 |
| Dihydrofolate reductase-like protein 1 | DHFRL1 |
| Disrupted in renal carcinoma 2 | DIRC2 |
| DNA excision repair protein ERCC-6 | ERCC6 |
| DNA mismatch repair protein Mlh3 (MutL protein homolog 3) | MLH3 |
| DNA polymerase delta subunit 4 | POLD4 |
| DNA-binding death effector domain-containing protein 2 | DEDD2 |
| F-box only protein 9 (NY-REN-57 antigen) | FBXO9 |
| Fibrinogen beta chain precursor | FGB |
| FK506-binding protein 3 | FKBP3 |
| FLJ16641 protein | LEKR1 |
| FLJ34870 protein | NP_997364.2 |
| Galectin-3-binding protein precursor | LGALS3BP |
| Gamma-aminobutyric-acid receptor alpha-1 subunit precursor | GABRA1 |
| Germ cell associated 1 isoform 1 | GSG1 |
| Glucagon-like peptide 1 receptor precursor (GLP-1 receptor) | GLP1R |
| Glucosamine-6-phosphate isomerase | GNPDA1 |
| Glucosaminyl (N-acetyl) transferase 3 mucin type | GCNT3 |
| Glutathione S-transferase A1 | GSTA1 |
| Glycosyltransferase | C3orf39 |
| GMP reductase 1 | GMPR |
| Guanine nucleotide-binding protein G(I)/G(S)/G(O) gamma-12 s | GNG12 |
| Heat shock factor 2-binding protein | HSF2BP |
| Heat shock protein HSP 90-alpha (HSP 86) | HSP90AA1 |
| HECT domain containing 2 isoform b | HECTD2 |
| Hemoglobin delta subunit (Hemoglobin delta chain) | HBD |
| Hepatocellular carcinoma-associated antigen 127 | KIAA1166 |
| Hepatocyte growth factor precursor (Scatter factor) | HGF |
| Hepatocyte nuclear factor 1-alpha (HNF-1A) | TCF1 |
| HERV-F(c)1Xq21.33 provirus ancestral Env polyprotein precursor | EFC1 |
| Hexosaminidase | HEXDC |
| ICOS ligand precursor (B7 homolog 2) | ICOSLG |
| Melanin-concentrating hormone receptor 2 | MCHR2 |
| Membrane-associated RING finger protein 4 | 38780 |
| Mesoderm induction early response protein 1 | MIER1 |
| Mitochondrial import inner membrane translocase subunit | TIMM17A |
| Mixed lineage kinase domain-like | MLKL |
| Muscle-cadherin precursor (M-cadherin) (Cadherin-15) | CDH15 |
| Myotubularin-related protein 7 (EC 3.1.3.-). | MTMR7 |
| NALP1 protein | Q96AM0 |
| Neurolysin mitochondrial precursor | NLN |
| NifU-like N-terminal domain-containing protein | NIFUN |
| Nitric-oxide synthase IIC | NOS2C |
| No distinctive protein motifs; ORF | Q15288 |
| Nuclear pore membrane protein 121 | NP_742017.1 |
| Olfactory receptor 1S2. | OR1S2 |
| Olfactory receptor 4K17 | OR4K17 |
| Olfactory receptor 5V1 (Hs6M1-21) | OR5V1 |
| Olfactory receptor 8G1 (Olfactory receptor TPCR25) | OR8G1 |
| Olfactory receptor 8K5 | OR8K5 |
| Olfactory receptor, family 4, subfamily D, member 1 |  |
| Olfactory receptor, family 5, subfamily K, member 1 |  |
| Olfactory receptor, family 6, subfamily C, member 3 |  |
| Orexigenic neuropeptide QRFP | GPR103 |
| Orphan nuclear receptor NR6A1 | NR6A1 |
| Solute carrier family 6, member 15 | S6A15 |
| patatin-like phospholipase domain containing 5 | PNPLA5 |
| PDZ domain containing 5B (Fragment) | Q5RJ30 |
| Peptidyl-glycine alpha-amidating monooxygenase precursor | PAM |
| Peroxisomal proliferator-activated receptor A-interacting complex 285 | PR285 |
| Phospholipase C-like 1 | PLCL1 |
| Phospholipase C-like 3 | PLCH1 |
| Pituitary tumor-transforming 2 |  |
| Plakophilin-4 (p0071) | PKP4 |
| Platelet factor 4 variant precursor (PF4var1) | PF4V1 |
| PLEKHM2 protein (Fragment) | PLEKHM2 |
| Polycystic kidney disease 1-like isoform b | KIAA0319L |
| Polypyrimidine tract-binding protein 2 | PTBP2 |
| Potassium voltage-gated channel subfamily A member 5 (Voltag | KCNA5 |
| Potassium voltage-gated channel subfamily KQT member 5 (Volt | KCNQ5 |
| Potassium voltage-gated channel, Shaw-related subfamily |  |
| Pre-B-cell leukemia transcription factor 2 |  |
| PREDICTED: similar to ankyrin repeat domain 26 isoform 1 | XP_292717.5 |
| PREDICTED: similar to Zinc finger protein 479 | XP_943217.1 |
| Pregnancy-specific beta-1-glycoprotein 9 precursor | PSG9 |
| Probable nucleolar complex protein 14 | NOP14 |
| Progesterone receptor (PR) | PGR |
| Prolactin receptor precursor (PRL-R) | PRLR |
| Protein C19orf18 precursor | C19orf18 |
| Protein disulfide-isomerase A3 precursor | PDIA3 |
| Protein FAM13A1 | FAM13A1 |
| Protein piccolo (Aczonin) | PCLO |
| Protein PRO0461 | P461 |
| Protein PRO0461 | P461 |
| Protein tyrosine phosphatase-like member A | PTPLA |
| Protein Wnt-7b precursor | WNT7B |
| Protein-arginine deiminase type-4 | PADI4 |
| Protein-glutamine gamma-glutamyltransferase 6 | TGM6 |
| Protocadherin beta 13 precursor | PCDHB13 |
| PTPN13-like protein Y-linked | PRY|PRY2 |
| Pumilio homolog 2 (Pumilio-2) | PUM2 |
| Putative GTP-binding protein RAY-like (Rab-like protein 4). | RABL4 |
| Putative mucin core protein 24 precursor | CD164 |
| Pyrroline-5-carboxylate reductase-like | PYCRL |
| RAB10, member RAS oncogene family |  |
| Radical S-adenosyl methionine domain containing 1 | RSAD1 |
| Ral guanine nucleotide dissociation stimulator-like 1 | RGL1 |
| Ras GTPase-activating protein 2 (GAP1m) | RASA2 |
| Ras GTPase-activating protein SynGAP | ZBTB9 |
| Ras homolog gene family member T1 isoform 2 | RHOT1 |
| Ras-related protein Rab-27B (C25KG). | RAB27B |
| Receptor-type tyrosine-protein phosphatase O precursor | PTPRO |
| Regenerating islet-derived protein 3 alpha precursor | REG3A |
| Related to CPSF subunits 68 kDa isoform 1 | CPSF3L |
| Rho GTPase activating protein 10 | ARHGAP10 |
| RNA polymerase II subunit A C-terminal domain phosphatase | CTDP1 |
| S-arrestin (Retinal S-antigen) | SAG |
| Scm-like with four MBT domains protein 2 | SMBT2 |
| Semaphorin-6B precursor (Semaphorin Z) (Sema Z) | SEMA6B |
| Semenogelin-1 precursor (Semenogelin I) (SGI) | SEMG1 |
| Serologically defined colon cancer antigen 8 | SDCCAG8 |
| Seven transmembrane helix receptor | Q8NH11 |
| Signal peptidase complex subunit 2 homolog | SPCS2 |
| Similar to AVLV472 | NP_996849.1 |
| Similar to chromosome 9 open reading frame 36 | XR_000527.1 |
| Similar to Olfactory receptor 56A4 |  |
| Similar to Ribonuclease H1 |  |
| Small EDRK-rich factor 1a. | SERF1A |
| Small inducible cytokine A25 precursor (CCL25) | CCL25 |
| Small inducible cytokine B6 precursor (CXCL6) | CXCL6 |
| SNF-related serine/threonine-protein kinase | SNRK |
| Sno strawberry notch homolog 1 | SBNO1 |
| Solute carrier family 22 member 1 isoform b | SLC22A1 |
| Solute carrier family 7 | SLC7A13 |
| Sp110 nuclear body protein (Speckled 110 kDa) | SP110 |
| Speckle-type POZ protein | SPOP |
| SSXT protein (Synovial sarcoma translocated to X chromosome) | SS18 |
| Stonin-1 (Stoned B-like factor) | STON1 |
| Structural maintenance of chromosome 2-like 1 protein | SMC2L1 |
| Synaptic vesicle protein 2B homolog | SV2B |
| synaptonemal complex central element protein 1 isoform 1 | SYCE1 |
| Taste receptor type 2 member 55 (T2R55) | TAS2R42 |
| Testis-specific serine/threonine-protein kinase 4 | TSSK4 |
| TGFB1-induced anti-apoptotic factor 1 |  |
| THAP domain-containing protein 11 | THAP11 |
| Thioredoxin domain-containing protein 11 | TXNDC11 |
| Transmembrane emp24 domain-containing protein 5 precursor | TMED5 |
| Transmembrane protease serine 13 | TMPRSS13 |
| Transmembrane protein 112 | TMEM112 |
| tRNA (adenine-N(1)-)-methyltransferase non-catalytic subunit | TRM6 |
| Tryptophan 5-hydroxylase 1 | TPH1 |
| Tubulin alpha-8 chain (Alpha-tubulin 8) | TBA8 |
| Twinfilin-2 (Twinfilin-1-like protein) (A6-related protein) | PTK9L |
| Tyrosine-protein kinase BTK | BTK |
| Ubiquitin carboxyl-terminal hydrolase 6 | UBP6 |
| Ubiquitin conjugation factor E4 A | UBE4A |
| Vacuolar ATP synthase subunit G 3 | ATP6V1G3 |
| Voltage-dependent calcium channel gamma-5 subunit | CCG5 |
| WD repeat SAM and U-box domain containing 1 | WDSUB1 |
| WD repeat domain 61 | WDR61 |
| Xaa-Pro aminopeptidase 2 precursor | XPNPEP2 |
| Zinc finger protein subfamily 1A, 5 | ZNFN1A5 |
| Zinc finger protein 14 | ZNF14 |
| Zinc finger protein 181 (HHZ181) | ZNF181 |
| Zinc finger protein 208 | ZNF208 |
| Zinc finger protein 23 | ZNF23 |
| Zinc finger protein 253 |  |
| Zinc finger protein 267 (Zinc finger protein HZF2) | ZNF267 |
| Zinc finger protein 311 | ZNF311 |
| Zinc finger protein 334 | ZNF334 |
| Zinc finger protein 342 | ZNF342 |
| Zinc finger protein 347 (Zinc finger 1111) | ZNF347 |
| Zinc finger protein 452 | ZNF452 |
| Zinc finger protein 548 | ZNF548 |
| Zinc finger protein 560 | ZNF560 |
| Zinc finger protein 573 | ZNF573 |
| Zinc finger protein 578 | ZN578 |
| Zinc finger protein 594 |  |
| Zinc finger protein 600 | ZNF600 |
| Zinc finger protein 625 | ZNF625 |
| Zinc finger protein 642 | ZNF642 |
| Zinc finger protein 658 | ZNF658 |
| Zinc finger protein 676 | ZN676 |
| Zinc finger protein 679 | ZNF679 |
| Zinc finger protein 681 | ZNF681 |
| Zinc finger protein 684 | ZNF684 |
| Zinc finger protein 708 (Zinc finger protein 15-like 1) | ZNF708 |
| Zinc finger protein 721 |  |
| Zinc finger protein 780B | ZNF780B |
| Zinc finger protein LOC653284 |  |
| Zinc finger-like transcript variant 2 | XR_001415.1 |
| Zinc transporter 1 (ZnT-1) | SLC30A1 |

**Table S2. Cellular genes upregulated in ORF3-expressing cells**

| Gene Name | Gene Symbol |
| --- | --- |
| Protein phosphatase 2C | PPM2C |
| Receptor-associated protein of the synapse | RAPSN |
| Abhydrolase domain-containing protein 2 | ABHD2 |
| Alpha-actin-1 | ACTA1 |
| Acyl-coenzyme A thioesterase 2 | ACOT2 |
| ADAMTS-1 precursor | ADAMTS1 |
| Adenosine kinase | ADK |
| ADP-ribosylation factor-like protein 8B | ARL8B |
| Agouti signaling protein precursor | ASIP |
| Alpha-endosulfine (ARPP-19e) | ENSA |
| Ankyrin repeat SAM and basic leucine zipper domain-containing 1 | ASZ1 |
| Apical-like protein | APXL |
| Archaemetzincin-1 | AMZ1 |
| Ataxin-7 | ATX7 |
| ATP/GTP binding protein-like 2 | NP_079059.2 |
| ClpX caseinolytic peptidase X homolog | CLPX |
| Biogenesis of lysosome-related organelles complex-1 subunit 2 | BLOC1S2 |
| Chloride channel protein skeletal muscle | CLCN1 |
| Chromosome 1 open reading frame 156 | C1orf156 |
| Coagulation factor IX precursor | F9 |
| Collagen alpha-1(XIV) chain precursor (Undulin) | COEA1 |
| COMM domain-containing protein 3 | COMMD3 |
| Coronin-1A (Coronin-like protein p57) | CORO1A |
| Cutaneous T-cell lymphoma tumor antigen se70-2 | C13orf10 |
| Cytochrome c oxidase polypeptide VIIa | COX7A1 |
| Delta-type opioid receptor | OPRD1 |
| Desmoglein-3 precursor | DSG3 |
| Dihydropyridine-sensitive L-type calcium channel alpha-2/de | CACNA2D1 |
| DKFZP434B0335 protein |  |
| DNA cross-link repair 1B protein (hSNM1B) | DCLRE1B |
| DNA repair protein REV1 | REV1L |
| DPCD protein | NP_056263.1 |
| FL cytokine receptor precursor | FLT3 |
| FLJ43505 protein | NP_997351.1 |
| Forkhead box K1 | NP_001032242.1 |
| Frizzled 6 precursor (Frizzled-6) (Fz-6) (hFz6) | FZD6 |
| Gamma-tubulin complex component 5 (GCP-5) | TUBGCP5 |
| Gastrin/cholecystokinin type B receptor (CCK-B receptor) | CCKBR |
| GDNF family receptor alpha-1 precursor (GFR-alpha-1) | GFRA1 |
| Gephyrin | GPHN |
| Glutathione-requiring prostaglandin D synthase | PTGD2 |
| Glycoprotein Xg precursor | XG |
| Colony stimulating factor 2 receptor, alpha, low-affinity (granulocyte-macrophage) | CSF2RA |
| GTPase IMAP family member 6 isoform 1 | GIMAP6 |
| Heparan sulfate glucosamine 3-O-sulfotransferase 2 | HS3ST2 |
| Heparanase precursor | HPSE |
| Melanoma-associated antigen B3 (MAGE-B3 antigen) | MAGEB3 |
| Metabotropic glutamate receptor 6 precursor (mGluR6) | GRM6 |
| MIR-interacting saposin-like protein precursor | TMEM4 |
| Myocyte-specific enhancer factor 2A | MEF2A |
| Natural killer cell receptor 2B4 precursor (NKR2B4) | CD244 |
| Natural killer cell-specific antigen KLIP1 | YIPF3 |
| Neprilysin | MME |
| Nervous system abundant protein 11 | Q86YR2 |
| Nuclear factor of kappa light polypeptide gene enhancer in B | NP_113607.1 |
| Nuclear transcription factor Y subunit gamma | NFYC |
| Nucleoside diphosphate kinase homolog 5 | NME5 |
| NudC domain-containing protein 2 | NUDCD2 |
| Oligopeptide transporter small intestine isoform | SLC15A1 |
| Oxytocin-neurophysin 1 precursor (OT-NPI) | OXT |
| P2X purinoceptor 6 (ATP receptor) (P2X6) | P2RXL1 |
| P2Y purinoceptor 1 (ATP receptor) (P2Y1) | P2RY1 |
| Phosphatidylinositol-3,4,5-trisphosphate-dependent Rac exchange factor 1 | PREX1 |
| Phospholipase D2 | PLD2 |
| Plexin domain-containing protein 2 precursor | PLXDC2 |
| Pogo transposable element with ZNF domain | POGZ |
| Polypeptide N-acetylgalactosaminyltransferase 14 | GALNT14 |
| PREDICTED: similar to RAN-binding protein 2-like 1 isoform 1 | XP_935024.1 |
| Probable G-protein coupled receptor 55 | GPR55 |
| Probable phospholipid-transporting ATPase ID | ATP8B2 |
| Protein C14orf108 | CN108 |
| Protein CutA precursor | CUTA |
| Protein NipSnap2 (Glioblastoma amplified sequence) | GBAS |
| Natural killer cell protein 7 | NKG7 |
| Protein PP2447 | YV03 |
| Putative helicase MOV-10 | MOV10 |
| Putative RNA-binding protein 11 (RNA-binding motif protein 1 | RBM11 |
| Putative UST1-like organic anion transporter | NP_955384.2 |
| Regulator of G-protein signaling 20 (RGS20) | RGS20 |
| Retinoschisin precursor | RS1 |
| Rhabdoid tumor deletion region protein 1 | RTDR1 |
| Ribosomal protein S6 kinase alpha-4 | RPS6KA4 |
| RNA binding motif single stranded interacting protein 3 | RBMS3 |
| Semaphorin-3F precursor | SEMA3F |
| SH2B adaptor protein 2 |  |
| Signal recognition particle 9 kDa protein | SRP9 |
| Similar to Brain-specific Na-dependent inorganic phosphate c |  |
| Similar to DFS70 (Fragment). |  |
| Sorting nexin-10 | SNX10 |
| Stereocilin precursor | STRC |
| SUDS3 protein | Q52LB7 |
| Synaptonemal complex protein 1 | SYCP1 |
| Synaptotagmin-14 | SYT14 |
| TBC1 domain family member 7 | TBC1D7 |
| Thioredoxin-like protein 4A | TXNL4A |
| Transmembrane gamma-carboxyglutamic acid protein 2 precursor | PRRG2 |
| Transmembrane protease serine 9 | TMPRSS9 |
| Transmembrane protein 106C | TMEM106C |
| Transmembrane protein 125 | TMEM125 |
| Transmembrane protein 160 | NP_060324.1 |
| Transmembrane protein 16G isoform NGEP long | TMEM16G |
| Transmembrane protein 46 precursor. | TMEM46 |
| tRNA-(N1G37) methyltransferase | NP_065861.1 |
| Troponin T slow skeletal muscle (TnTs) | TNNT1 |
| Tubby-related protein 2 | TULP2 |
| ULK4 protein | Q9UF96 |
| Uroplakin-1a | UPK1A |
| Vitronectin precursor | VTN |
| Voltage-dependent calcium channel gamma-2 subunit | CACNG2 |
| Voltage-dependent L-type calcium channel beta-4 subunit | CACNB4 |
| WD repeat and HMG-box DNA-binding protein 1 | WDHD1 |
| WD-repeat protein 33 (WD-repeat protein WDC146) | WDR33 |
| Zinc finger protein 14 homolog (Zfp-14 | ZFP14 |
| Zinc finger protein 532 | ZNF532 |
| Zinc finger protein 536 | ZNF536 |

**Table S3. RT-PCR primers**

| **Gene** | **Forward Primer** | **Reverse Primer** | **Product Size** |
| --- | --- | --- | --- |
| Hexon | GTGTTGTAGGCAGTGCCGGAGTAGGG | CCTACGCACGATGTGACCACAGACCG | 215 bp |
| ATF1 | GAAGATTCCCACAAGAGTACCAC | GCCTATGCTGTCGGATGAGTC | 144 bp |
| CD72 | GAAGCACTACAGGTGGAACAG | CCGCATGTGAAGAAGGGCT | 176 bp |
| MAOA | TGAGCGTCTCGTTCAATATGTC | CATCAGTTGGAATCTCCTTCCC | 143 bp |
| TCF1 | CGGAGGAACCGTTTCAAGTG | GCATTCCGCCCTATTGCAC | 126 bp |
| RASA2 | AGTCCAGTGGTACGAGTGAG | ATCTTTGCCTGGCTGTTTGTG | 155 bp |
| ATP5J | GTTCTCCTCTGTCATTCGGTCA | CCAGCTCTTGCTGATACTCTGAA | 191 bp |
| SP110 | TCGGAATGAGGATGGAACTTGG | CAGAGCAAAAGTCCACTCTTCAG | 141 bp |
| POLD4 | ATCACTGATTCCTACCCGGTT | AGAGATGCCAGAGACTGCACT | 295 bp |
| Histone H4 | TGAGAGACAACATTCAGGGCATCAC | CGCTTGAGCGCGTACACCACATCCAT | 211 bp |

Annealing Temperature: 55ºC

Amplification cycles: 30 to 35
