# Supplementary material for: The Hepatitis E Virus ORF3 Protein Regulates the Expression of Liver-Specific Genes by Modulating Localization of Hepatocyte Nuclear Factor 4
Source: PLoS One. 2011 Jul 20;6(7):e22412. doi: 10.1371/journal.pone.0022412 (PMC3140526; doi:10.1371/journal.pone.0022412)
Supplement: Table S2 — Cellular genes upregulated in ORF3-expressing cells. (DOC) [file pone.0022412.s003.doc]

**Table S2. Cellular genes upregulated in ORF3-expressing cells**

| Gene Name | Gene Symbol |
| --- | --- |
| Protein phosphatase 2C | PPM2C |
| Receptor-associated protein of the synapse | RAPSN |
| Abhydrolase domain-containing protein 2 | ABHD2 |
| Alpha-actin-1 | ACTA1 |
| Acyl-coenzyme A thioesterase 2 | ACOT2 |
| ADAMTS-1 precursor | ADAMTS1 |
| Adenosine kinase | ADK |
| ADP-ribosylation factor-like protein 8B | ARL8B |
| Agouti signaling protein precursor | ASIP |
| Alpha-endosulfine (ARPP-19e) | ENSA |
| Ankyrin repeat SAM and basic leucine zipper domain-containing 1 | ASZ1 |
| Apical-like protein | APXL |
| Archaemetzincin-1 | AMZ1 |
| Ataxin-7 | ATX7 |
| ATP/GTP binding protein-like 2 | NP_079059.2 |
| ClpX caseinolytic peptidase X homolog | CLPX |
| Biogenesis of lysosome-related organelles complex-1 subunit 2 | BLOC1S2 |
| Chloride channel protein skeletal muscle | CLCN1 |
| Chromosome 1 open reading frame 156 | C1orf156 |
| Coagulation factor IX precursor | F9 |
| Collagen alpha-1(XIV) chain precursor (Undulin) | COEA1 |
| COMM domain-containing protein 3 | COMMD3 |
| Coronin-1A (Coronin-like protein p57) | CORO1A |
| Cutaneous T-cell lymphoma tumor antigen se70-2 | C13orf10 |
| Cytochrome c oxidase polypeptide VIIa | COX7A1 |
| Delta-type opioid receptor | OPRD1 |
| Desmoglein-3 precursor | DSG3 |
| Dihydropyridine-sensitive L-type calcium channel alpha-2/de | CACNA2D1 |
| DKFZP434B0335 protein |  |
| DNA cross-link repair 1B protein (hSNM1B) | DCLRE1B |
| DNA repair protein REV1 | REV1L |
| DPCD protein | NP_056263.1 |
| FL cytokine receptor precursor | FLT3 |
| FLJ43505 protein | NP_997351.1 |
| Forkhead box K1 | NP_001032242.1 |
| Frizzled 6 precursor (Frizzled-6) (Fz-6) (hFz6) | FZD6 |
| Gamma-tubulin complex component 5 (GCP-5) | TUBGCP5 |
| Gastrin/cholecystokinin type B receptor (CCK-B receptor) | CCKBR |
| GDNF family receptor alpha-1 precursor (GFR-alpha-1) | GFRA1 |
| Gephyrin | GPHN |
| Glutathione-requiring prostaglandin D synthase | PTGD2 |
| Glycoprotein Xg precursor | XG |
| Colony stimulating factor 2 receptor, alpha, low-affinity (granulocyte-macrophage) | CSF2RA |
| GTPase IMAP family member 6 isoform 1 | GIMAP6 |
| Heparan sulfate glucosamine 3-O-sulfotransferase 2 | HS3ST2 |
| Heparanase precursor | HPSE |
| Melanoma-associated antigen B3 (MAGE-B3 antigen) | MAGEB3 |
| Metabotropic glutamate receptor 6 precursor (mGluR6) | GRM6 |
| MIR-interacting saposin-like protein precursor | TMEM4 |
| Myocyte-specific enhancer factor 2A | MEF2A |
| Natural killer cell receptor 2B4 precursor (NKR2B4) | CD244 |
| Natural killer cell-specific antigen KLIP1 | YIPF3 |
| Neprilysin | MME |
| Nervous system abundant protein 11 | Q86YR2 |
| Nuclear factor of kappa light polypeptide gene enhancer in B | NP_113607.1 |
| Nuclear transcription factor Y subunit gamma | NFYC |
| Nucleoside diphosphate kinase homolog 5 | NME5 |
| NudC domain-containing protein 2 | NUDCD2 |
| Oligopeptide transporter small intestine isoform | SLC15A1 |
| Oxytocin-neurophysin 1 precursor (OT-NPI) | OXT |
| P2X purinoceptor 6 (ATP receptor) (P2X6) | P2RXL1 |
| P2Y purinoceptor 1 (ATP receptor) (P2Y1) | P2RY1 |
| Phosphatidylinositol-3,4,5-trisphosphate-dependent Rac exchange factor 1 | PREX1 |
| Phospholipase D2 | PLD2 |
| Plexin domain-containing protein 2 precursor | PLXDC2 |
| Pogo transposable element with ZNF domain | POGZ |
| Polypeptide N-acetylgalactosaminyltransferase 14 | GALNT14 |
| PREDICTED: similar to RAN-binding protein 2-like 1 isoform 1 | XP_935024.1 |
| Probable G-protein coupled receptor 55 | GPR55 |
| Probable phospholipid-transporting ATPase ID | ATP8B2 |
| Protein C14orf108 | CN108 |
| Protein CutA precursor | CUTA |
| Protein NipSnap2 (Glioblastoma amplified sequence) | GBAS |
| Natural killer cell protein 7 | NKG7 |
| Protein PP2447 | YV03 |
| Putative helicase MOV-10 | MOV10 |
| Putative RNA-binding protein 11 (RNA-binding motif protein 1 | RBM11 |
| Putative UST1-like organic anion transporter | NP_955384.2 |
| Regulator of G-protein signaling 20 (RGS20) | RGS20 |
| Retinoschisin precursor | RS1 |
| Rhabdoid tumor deletion region protein 1 | RTDR1 |
| Ribosomal protein S6 kinase alpha-4 | RPS6KA4 |
| RNA binding motif single stranded interacting protein 3 | RBMS3 |
| Semaphorin-3F precursor | SEMA3F |
| SH2B adaptor protein 2 |  |
| Signal recognition particle 9 kDa protein | SRP9 |
| Similar to Brain-specific Na-dependent inorganic phosphate c |  |
| Similar to DFS70 (Fragment). |  |
| Sorting nexin-10 | SNX10 |
| Stereocilin precursor | STRC |
| SUDS3 protein | Q52LB7 |
| Synaptonemal complex protein 1 | SYCP1 |
| Synaptotagmin-14 | SYT14 |
| TBC1 domain family member 7 | TBC1D7 |
| Thioredoxin-like protein 4A | TXNL4A |
| Transmembrane gamma-carboxyglutamic acid protein 2 precursor | PRRG2 |
| Transmembrane protease serine 9 | TMPRSS9 |
| Transmembrane protein 106C | TMEM106C |
| Transmembrane protein 125 | TMEM125 |
| Transmembrane protein 160 | NP_060324.1 |
| Transmembrane protein 16G isoform NGEP long | TMEM16G |
| Transmembrane protein 46 precursor. | TMEM46 |
| tRNA-(N1G37) methyltransferase | NP_065861.1 |
| Troponin T slow skeletal muscle (TnTs) | TNNT1 |
| Tubby-related protein 2 | TULP2 |
| ULK4 protein | Q9UF96 |
| Uroplakin-1a | UPK1A |
| Vitronectin precursor | VTN |
| Voltage-dependent calcium channel gamma-2 subunit | CACNG2 |
| Voltage-dependent L-type calcium channel beta-4 subunit | CACNB4 |
| WD repeat and HMG-box DNA-binding protein 1 | WDHD1 |
| WD-repeat protein 33 (WD-repeat protein WDC146) | WDR33 |
| Zinc finger protein 14 homolog (Zfp-14 | ZFP14 |
| Zinc finger protein 532 | ZNF532 |
| Zinc finger protein 536 | ZNF536 |

**Table S3. RT-PCR primers**

| **Gene** | **Forward Primer** | **Reverse Primer** | **Product Size** |
| --- | --- | --- | --- |
| Hexon | GTGTTGTAGGCAGTGCCGGAGTAGGG | CCTACGCACGATGTGACCACAGACCG | 215 bp |
| ATF1 | GAAGATTCCCACAAGAGTACCAC | GCCTATGCTGTCGGATGAGTC | 144 bp |
| CD72 | GAAGCACTACAGGTGGAACAG | CCGCATGTGAAGAAGGGCT | 176 bp |
| MAOA | TGAGCGTCTCGTTCAATATGTC | CATCAGTTGGAATCTCCTTCCC | 143 bp |
| TCF1 | CGGAGGAACCGTTTCAAGTG | GCATTCCGCCCTATTGCAC | 126 bp |
| RASA2 | AGTCCAGTGGTACGAGTGAG | ATCTTTGCCTGGCTGTTTGTG | 155 bp |
| ATP5J | GTTCTCCTCTGTCATTCGGTCA | CCAGCTCTTGCTGATACTCTGAA | 191 bp |
| SP110 | TCGGAATGAGGATGGAACTTGG | CAGAGCAAAAGTCCACTCTTCAG | 141 bp |
| POLD4 | ATCACTGATTCCTACCCGGTT | AGAGATGCCAGAGACTGCACT | 295 bp |
| Histone H4 | TGAGAGACAACATTCAGGGCATCAC | CGCTTGAGCGCGTACACCACATCCAT | 211 bp |

Annealing Temperature: 55ºC

Amplification cycles: 30 to 35
