# Supplementary material for: The Hepatitis E Virus ORF3 Protein Regulates the Expression of Liver-Specific Genes by Modulating Localization of Hepatocyte Nuclear Factor 4
Source: PLoS One. 2011 Jul 20;6(7):e22412. doi: 10.1371/journal.pone.0022412 (PMC3140526; doi:10.1371/journal.pone.0022412)
Supplement: Table S3 — RT-PCR primers. (DOC) [file pone.0022412.s004.doc]

**Table S3. RT-PCR primers**

| **Gene** | **Forward Primer** | **Reverse Primer** | **Product Size** |
| --- | --- | --- | --- |
| Hexon | GTGTTGTAGGCAGTGCCGGAGTAGGG | CCTACGCACGATGTGACCACAGACCG | 215 bp |
| ATF1 | GAAGATTCCCACAAGAGTACCAC | GCCTATGCTGTCGGATGAGTC | 144 bp |
| CD72 | GAAGCACTACAGGTGGAACAG | CCGCATGTGAAGAAGGGCT | 176 bp |
| MAOA | TGAGCGTCTCGTTCAATATGTC | CATCAGTTGGAATCTCCTTCCC | 143 bp |
| TCF1 | CGGAGGAACCGTTTCAAGTG | GCATTCCGCCCTATTGCAC | 126 bp |
| RASA2 | AGTCCAGTGGTACGAGTGAG | ATCTTTGCCTGGCTGTTTGTG | 155 bp |
| ATP5J | GTTCTCCTCTGTCATTCGGTCA | CCAGCTCTTGCTGATACTCTGAA | 191 bp |
| SP110 | TCGGAATGAGGATGGAACTTGG | CAGAGCAAAAGTCCACTCTTCAG | 141 bp |
| POLD4 | ATCACTGATTCCTACCCGGTT | AGAGATGCCAGAGACTGCACT | 295 bp |
| Histone H4 | TGAGAGACAACATTCAGGGCATCAC | CGCTTGAGCGCGTACACCACATCCAT | 211 bp |

Annealing Temperature: 55ºC

Amplification cycles: 30 to 35
